# The relationship between physical activity and work engagement among university teachers: the chain mediating effect of resilience and cognitive flexibility

**DOI:** 10.3389/fpsyg.2026.1866338

**Published:** 2026-06-19

**Authors:** Fang Wang

**Affiliations:** School of Sports Training, Chengdu Sports University, Chengdu, Sichuan Province, China

**Keywords:** cognitive flexibility, physical activity, resilience, university teachers, work engagement

## Abstract

**Objective:**

To examine the relationship between physical activity and work engagement among university teachers, analyze the chained mediating roles of resilience and cognitive flexibility in this relationship, and provide a theoretical basis and practical guidance for enhancing the mental health and work efficacy of university teachers.

**Methods:**

A convenience sampling method was used to select 500 in-service teachers from multiple universities in Sichuan Province as participants. The study employed the Physical Activity Rating Scale-3 (PARS-3), the Connor-Davidson Resilience Scale (CD-RISC), the Cognitive Flexibility Inventory (CFI), and the Utrecht Work Engagement Scale (UWES) for questionnaire surveys. A total of 378 valid questionnaires were collected from university teachers (173 males, 205 females; mean age = 24.6 ± 11.2 years). Descriptive statistics, correlation analysis, and chain mediating effect testing (Bootstrap method) were conducted using SPSS 24.0 and the PROCESS macro (Model 6).

**Results:**

(1) Correlation analysis showed significant positive correlations among university teachers’ physical activity, resilience, cognitive flexibility, and work engagement (*p* < 0.01). (2) Structural equation modeling and mediating effect tests indicated that physical activity had a significant positive predictive effect on work engagement (*β* = 0.0066, 95% CI: [0.0037–0.0096]). (3) Mediating effect analysis revealed that resilience and cognitive flexibility not only played partial mediating roles separately but also jointly formed a chain mediating pathway between physical activity and work engagement.

**Conclusion:**

Physical activity among university teachers is not only directly and positively associated with their work engagement but also indirectly associated with higher work engagement through a sequential pathway involving resilience and then cognitive flexibility.

## 1 Introduction

Against the backdrop of continuous reform of the higher education system, university teachers, as the core agents of knowledge production, academic innovation, and talent cultivation, have their work status not only directly determining the quality of teaching and the level of research output but also profoundly affecting the sustainable competitiveness of higher education institutions and the national innovation-driven development strategy (Gonzales, 2012; O'Meara et al., 2014). Work engagement, as a core construct in the fields of positive organizational behavior and occupational health psychology, is defined as a positive, fulfilling, work-related psychological state, characterized by three dimensions: vigor, dedication, and absorption (Bakker et al., 2014). A substantial body of empirical research has shown a robust positive association between high levels of work engagement and teachers’ occupational well-being (Chen, 2025), organizational commitment (Ertas and Ozdemir, 2024), teaching efficacy (Xiao et al., 2022), and research productivity (Bakker and Bal, 2010).

However, in contrast, university teachers in recent years have faced increasing occupational stress and multiple role conflicts. Issues such as work-life imbalance, emotional exhaustion, and burnout have become increasingly prominent, posing a deep-seated concern that constrains the improvement of higher education quality (Artemiou et al., 2018; Wiegel et al., 2016). Recent research has also linked such occupational stress to emerging phenomena like “quiet quitting,” characterized by disengagement and reduced initiative (Dilekci et al., 2025). A study of university teachers in Portugal found that their overall level of work engagement is generally in the lower-middle range and is approaching a threshold that could pose a substantial threat to their physical and mental health (de Lourdes Machado-Taylor et al., 2016). In the Chinese context, university teachers face the superimposed effects of multiple pressures, including teaching load assessment, research performance evaluation, professional title promotion competition, and administrative affairs. The current situation of emotional exhaustion and insufficient work engagement is equally concerning (Li and Khattak, 2023; Yuan et al., 2022). Therefore, exploring the mechanisms that are associated with work engagement among university teachers and the factors that protect it—and identifying intervention pathways to effectively predict their work engagement from a positive psychology perspective—will contribute to the sustainable development of higher education.

Physical activity (PA), as a systematic, planned form of physical exertion aimed at improving or maintaining physical and mental functions, has been widely validated by interdisciplinary research for its positive benefits on individual physiological health and psychological functioning (Bull et al., 2020; Teixeira et al., 2012). In recent years, the spillover effects of physical exercise have attracted the attention of researchers in organizational management, specifically, whether and how the physical and mental benefits generated by physical exercise can “cross” the boundary of the life domain and translate into positive work states and performance in the workplace (Yang et al., 2026). Based on the effort-recovery model, Xu (2019) found that daily physical exercise indirectly promotes next-day in-role job performance and extra-role organizational citizenship behavior by enhancing next-day morning positive affect and work engagement, and this gain effect was more pronounced on workdays when employees faced high job demands (Xu, 2019). Moreover, from the perspective of the conservation of resources (COR) theory, physical exercise can be understood as an active resource investment behavior occurring in the non-work domain. By investing time and energy in physical exercise, individuals accumulate valuable psychological resources such as positive affect and self-efficacy. Due to the cross-domain transfer ability of these resources, they can be mobilized and redeployed in the work context, thereby empowering work performance (Hagger, 2015; Jin et al., 2018). Some studies suggest that regular, long-term physical exercise can prolong and amplify the positive effects of remote work on self-control, thereby effectively enhancing employees’ work engagement (Paluszak et al., 2025; Wells et al., 2023). It is worth noting that although the association between physical exercise and work engagement has been preliminary validated among the general employee population, research systematically examining the facilitate mechanisms and underlying psychological processes of physical exercise on work engagement specifically among university teachers as a distinct occupational group remains limited (Jones et al., 2023; Munir et al., 2015). Therefore, based on the above theoretical and empirical evidence, this study proposes:

Hypothesis 1: Physical activity behavior among university teachers has a significant positive predictive effect on their level of work engagement.

Resilience, as an important construct in positive psychology and psychological capital theory, refers to the psychological ability and dynamic process through which individuals effectively cope with, quickly recover from, and positively adapt to adversity, trauma, threats, or significant sources of stress (Fletcher and Sarkar, 2013). Reinforcing this, a recent study demonstrated that resilience serves as a key mediator in the relationship between occupational stress and subjective well-being among teachers (Dilekci et al., 2025). In the workplace context, resilience is regarded as a crucial personal resource that helps employees buffer the erosive effects of occupational stress on physical and mental health, and maintain or restore positive work states (Hartmann et al., 2020; Ungar and Theron, 2020). According to attentional control theory, individuals with high resilience can more effectively regulate negative emotions under pressure, reducing the consumption of cognitive resources by emotions, thereby freeing up more working memory and executive function resources to support flexible thinking (Eysenck and Derakshan, 2011).

A cross-sectional study by Abdelhadi on engineers clearly indicated that resilience significantly promotes work engagement and job satisfaction, suggesting that organizations should cultivate employee resilience as an important strategy for creating a positive work environment and enhancing organizational effectiveness (Ibrahim and Hussein, 2024). Research on corporate employees has also found that resilience plays a partial mediating role between burnout and work engagement; that is, employees with higher levels of resilience are able to maintain relatively better work engagement even when experiencing high levels of burnout (Nantsupawat et al., 2024). From the perspective of affect dynamics, a recent study using a daily affect diary scale found that trait resilience indirectly promotes employees’ work engagement by reducing affective inertia—that is, the auto-correlation and rigidity of affective states—and that this indirect effect is particularly pronounced under conditions of high perceived stress (Liu and Chen, 2025). These findings reveal the emotion regulation mechanism through which resilience facilitates work engagement. For university teachers, the nature of their profession determines that they continuously face multidimensional challenges such as research uncertainty, teaching evaluation pressure, and career development bottlenecks (Dost, 2024; Ross et al., 2024). Resilience, as a key psychological protective resource, helps teachers maintain positive psychological orientation and sustained work motivation in the face of these adversities (Galindo-Dominguez et al., 2020). Accordingly, this study proposes:

Hypothesis 2: University teachers’ resilience has a significant positive predictive effect on their level of work engagement.

Cognitive flexibility, as one of the core sub-components of executive function, refers to the ability of an individual to flexibly adjust cognitive strategies and shift thinking patterns according to changes in environmental demands, thereby adapting to novel situations (Orakci, 2021). In contemporary organizations, as the complexity and dynamism of work tasks continue to increase, cognitive flexibility, as a cognitive resource at the individual level, has gained growing attention from academia for its functional value in occupational adaptation and work effectiveness (Cheng et al., 2017; Duru and Soner, 2026). The negative impact of occupational stress on mental health outcomes, such as anxiety, is well-documented, and understanding protective cognitive factors is crucial (Cicek et al., 2026). Uhlig examined the effects of cognitive demands of flexible work practices on employees’ cognitive flexibility, work engagement, and fatigue. Their findings indicated that cognitive demands in flexible work contexts (e.g., planning working times, planning working locations, and coordinating with others), serving as challenge stressors, can effectively promote the development of employees’ cognitive flexibility and stimulate their work engagement. That is, increased cognitive flexibility helps employees experience higher levels of motivation and engagement at work (Uhlig et al., 2023). Furthermore, cognitive flexibility plays an important moderating role in the relationship between job autonomy and work-related well-being; high cognitive flexibility strengthens the indirect effect of job autonomy on well-being through the cognitive-affective involvement pathway (Ozgen and Tangor, 2022).

Theoretically, university teachers with higher levels of cognitive flexibility are more adept at efficiently switching between different academic tasks (e.g., allocating cognitive resources among teaching, research, and administrative duties) and are better able to shift their thinking patterns and seek alternative solutions when facing academic difficulties or research setbacks (Camadan et al., 2025). Consequently, they can reduce the frustration and burnout tendency caused by cognitive rigidity, and maintain sustained psychological investment in academic work (Avci, 2026). Based on this reasoning, this study proposes Hypothesis 3: University teachers’ cognitive flexibility has a significant positive predictive effect on their level of work engagement.

**Figure 1:**
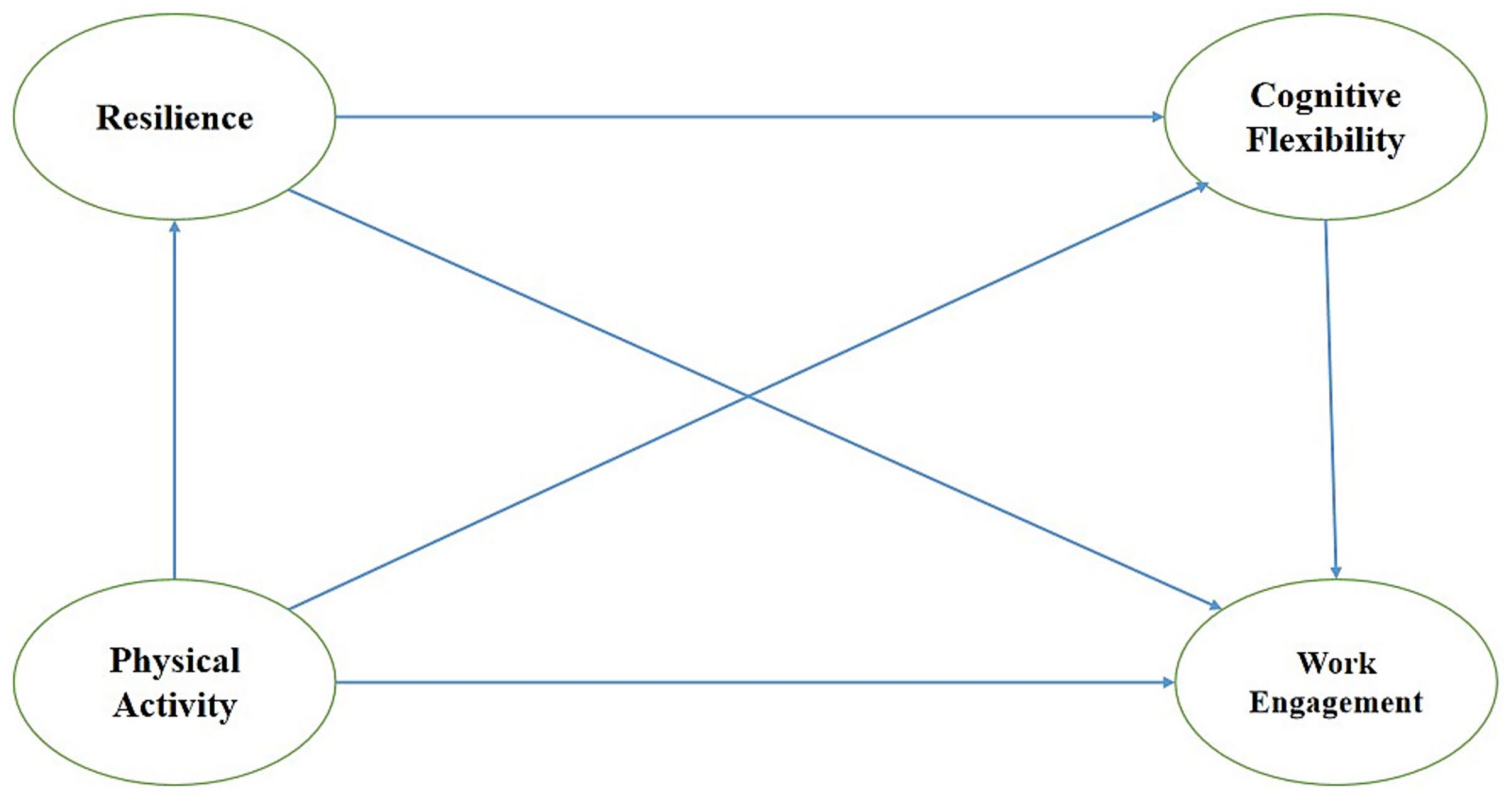
Hypothesis model.

Although the positive effects of physical exercise, psychological resilience, and cognitive flexibility on work engagement have each received a certain degree of theoretical support and empirical evidence (Jindo et al., 2020; Malik and Garg, 2020; Uhlig et al., 2023), there remains a lack of research that integrates these three factors into a single theoretical framework and systematically examines the psychological and cognitive pathways through which physical exercise indirectly influences work engagement. Therefore, based on the theoretical analysis presented above, the impact of physical exercise on university teachers members’ work engagement is not a simple direct effect or a single mediating process, but rather a complex pathway involving the interplay of multi-level psychological mechanisms, in which psychological resilience and cognitive flexibility likely serve as two key mediating variables that are hierarchically related (Kiema-Junes et al., 2022; Lopez-Canada et al., 2021). Based on the above logic, psychological resilience and cognitive flexibility likely form a chain-mediation framework in the process by which physical exercise influences work engagement: physical exercise first provides both physiological and psychological resources, and is associated with an individual’s level of resilience; the enhancement of psychological resilience, in turn, creates favorable internal psychological conditions for the development and application of cognitive flexibility, enabling individuals to maintain more flexible and adaptive cognitive patterns when facing complex work tasks; ultimately, this effect of increased psychological resources—driven by physical exercise and transmitted through psychological resilience and cognitive flexibility in successive stages—converges into higher levels of work engagement. Based on this, this study proposes Hypothesis 4: Psychological resilience and cognitive flexibility sequentially mediate (chain-mediate) the relationship between physical exercise and work engagement among university teachers. Specifically, the hypothesized pathway is: physical activity → psychological resilience → cognitive flexibility → work engagement. In other words, physical exercise is associated with higher levels of psychological resilience, which in turn is associated with greater cognitive flexibility, and this combined chain is ultimately associated with higher levels of work engagement (as shown in Figure 1).

## 2 Methods

### 2.1 Study population

This study employs a cross-sectional questionnaire survey design. Based on calculations of the estimated effect size using G*Power software, the minimum required sample size was determined to be 300, assuming a power of 0.80 and a significance level of *α* = 0.05 (Kang, 2021). To ensure sample representatives and allow for the exclusion of invalid questionnaires, the actual number of questionnaires distributed in this study was slightly higher than this minimum requirement. Convenience sampling was employed, utilizing a combination of online electronic and offline paper questionnaires. Surveys were distributed to full-time faculty members at multiple higher education institutions in Sichuan Province between September and December 2025. This study strictly adheres to the ethical guidelines of the Declaration of Helsinki and its subsequent amendments. The research protocol has been formally approved by the Ethics Committee of the School of Sports Training at Chengdu University of Physical Education, the authors’ affiliated institution (Ethics Approval No: CTYXLL2025006). Prior to distributing the questionnaires, researchers provided potential participants with clear and objective explanations of the study’s purpose, procedures, data anonymization methods, and their rights to voluntary participation and withdrawal at any time through standardized instructions. All questionnaires were completed independently by the faculty members after obtaining their informed consent.

A total of 500 questionnaires were distributed, with 421 returned. According to predefined data screening criteria, 43 invalid questionnaires were excluded due to repetitive responses, excessively short completion times, or missing more than 15% of key items. Ultimately, 378 valid questionnaires were obtained, resulting in a response rate of 84.2% and a validity rate of 89.79%. Among the 378 valid respondents, there were 173 male teachers and 205 female teachers. Specific demographic characteristics regarding the age distribution of respondents, average years of teaching experience, educational background, professional titles, nature of the institutions, and staffing status are detailed in Table 1.

**Table 1:** Descriptive statistics of the study population.

Project	Category	Number of people	Percentage
Gender	Male	173	45.77%
	Female	205	54.23%
Age	20–25	14	3.70%
	26–30	76	20.11%
	31–35	50	13.23%
	36–40	167	44.18%
	41 and older	71	18.78%
Years of teaching experience	1 year or less	5	1.32%
	2–5 years	25	6.61%
	6–10 years	66	17.46%
	11–15 years	205	54.23%
	>16 years	77	20.37%
Highest degree	Bachelor’s degree	21	5.56%
	Master’s degree	230	60.85%
	Doctorate	124	32.80%
Professional title	Lecturer	96	25.40%
	Associate Professor	116	30.69%
	Professor	94	24.87%
	None	72	19.05%
Type of institution	Public	323	85.45%
	Private	55	14.55%
Tenured/permanent faculty	Yes	295	78.04%
	No	83	21.96%

### 2.2 Measurement instruments

#### 2.2.1 Physical activity measurement scale

This study used the Physical Activity Rating Scale-3 (PARS-3) to assess university teachers’ level of physical activity participation. The scale was revised by Chinese scholar Liang (1994) based on the original version proposed by Japanese scholar Kenji Hashimoto in 1990, aiming to comprehensively evaluate an individual’s amount of physical activity from three dimensions: intensity, frequency, and duration. Each indicator of the scale is divided into five levels, rated on a 1 to 5 scale. The total amount of physical activity was calculated using the formula: Activity amount = Intensity × (Duration – 1) × Frequency, with scores ranging from 0 to 100. Higher scores indicate higher levels of individual physical activity. The classification criteria are as follows: total score ≤ 19 indicates low activity level, 20–42 indicates moderate activity level, and ≥ 43 indicates high activity level. In this study, the Cronbach’s *α* coefficient of this scale was 0.931.

#### 2.2.2 Resilience measurement scale

To measure university teachers’ resilience levels, this study used the Chinese version of the Connor-Davidson Resilience Scale (CD-RISC), developed by Connor and Davidson (2003) and revised by Yu Xiaonan and Zhang Jianxin. The scale consists of 25 items, covering three core dimensions: tenacity, strength, and optimism. Participants rated each item on a 5-point Likert scale ranging from 1 (“never”) to 5 (“always”). Higher total scores on the scale reflect greater prominence of resilience traits in respondents. In this study, the Cronbach’s *α* coefficient of this scale was 0.927.

#### 2.2.3 Cognitive flexibility measurement scale

This study used the Chinese version of the Cognitive Flexibility Inventory (CFI), developed by Dennis and Vander Wal and revised by Wu et al. (2017), to measure university teachers’ cognitive flexibility characteristics. The scale contains 20 items comprising two dimensions: the choice for cognitive flexibility and the tendency for cognitive flexibility. A sample item is “I am good at seeing difficult problems from different perspectives.” The scale uses a 5-point Likert scoring method, with higher scores indicating stronger ability to shift thinking and adjust strategies when facing cognitive challenges and situational changes. In this study, the Cronbach’s α coefficient of this scale was 0.909.

#### 2.2.4 Work engagement scale

This study used the Chinese version of the Utrecht Work Engagement Scale (UWES), developed by Schaufeli et al. (2019), to assess the level of work engagement among university teachers members. The scale consists of 15 items evenly distributed across three dimensions: vigor, dedication, and absorption. A sample item is “At my work, I feel bursting with energy. Participants responded on a 7-point Likert scale ranging from 1 (never) to 7 (always). Higher scores indicate deeper levels of work engagement. In this study, the Cronbach’s α coefficient of this scale was 0.920.

### 2.3 Data analysis

This study used SPSS 26.0 and its PROCESS macro for data management and statistical analysis (Hayes, 2018). The analysis procedure included the following steps. First, Harman’s one-factor test was used to examine common method bias. Second, descriptive statistics and Pearson correlation analyses were performed on the study variables to preliminarily reveal the trends of associations among the variables. Third, the bias-corrected nonparametric percentile Bootstrap method (with 5,000 resampling iterations) was used to test the chain mediation model. The 95% confidence intervals for the direct, indirect, and total effects were estimated using PROCESS Model 6. A confidence interval not containing zero indicated that the corresponding effect reached statistical significance. In addition, this study initially included gender, age, years of teaching experience, educational level, and professional title as control variables in the model analysis to account for their potential confounding effects. Preliminary regression analyses indicated that educational level and professional title were not significantly correlated with any of the core study variables (physical activity, psychological resilience, cognitive flexibility, and work engagement) (all *p* > 0.05), and their inclusion did not alter the direction, effect size, or significance of the hypothesized pathways. Prior to conducting the mediation analysis, the assumptions for linear regression (including linearity, independence of errors, homoscedasticity, and normality of residuals) were tested. No severe violations of these assumptions were found. Multicollinearity among the predictors was also examined using the variance inflation factor (VIF); all VIF values were below the conservative threshold of 5, indicating that there were no significant multicollinearity issues.

## 3 Results

### 3.1 Common method Bias

The study used Harman’s one-factor test to assess potential common method bias and reported the KMO value and Bartlett’s test of spherical results (Harman, 1976). The data showed a KMO value of 0.976, and Bartlett’s test of spherical yielded χ^2^ = 19,521.659, df = 1,953, *p* < 0.001, indicating that the correlation matrix among the variables was suitable for factor analysis (Podsakoff et al., 2003). Based on this, all measurement items were entered into an unrelated exploratory factor analysis. The results showed multiple factors with eigenvalues greater than 1, and the first factor accounted for only 32.63% of the total variance, which is below the recommended threshold of 40% (Podsakoff et al., 2003). To further rule out the influence of common method variance (CMV), we employed the unmeasured latent variable method factor approach (Podsakoff et al., 2003). We compared a measurement model in which all items were loaded onto their respective trait factors (five-factor model) with an additional model in which all items were loaded onto a single latent variable representing the common method factor. The inclusion of the common method factor did not significantly improve model fit (CFI < 0.01, RMSEA < 0.01), and the factor loadings of each item on the trait factors remained significant and substantial. These results indicate that CMV does not pose a major threat to the validity of the conclusions of this study. Accordingly, it can be concluded that serious common method bias is not present in this study, and the influence of common method variance is relatively small, rendering the subsequent analyses credible.

### 3.2 Correlation analysis

Table 2 presents the descriptive statistics and correlation coefficients for the study variables. The data showed that physical activity was significantly positively correlated with resilience (*r* = 0.396, *p* < 0.01), cognitive flexibility (*r* = 0.363, *p* < 0.01), and work engagement (*r* = 0.401, *p* < 0.01). Resilience was significantly positively correlated with cognitive flexibility (*r* = 0.420, *p* < 0.01) and work engagement (*r* = 0.431, *p* < 0.01). Cognitive flexibility was significantly positively correlated with work engagement (*r* = 0.435, *p* < 0.01). Overall, these coefficients indicate that all main variables exhibited pairwise positive associations at the same measurement time point. This pattern of covariation among the variables, as revealed by the correlation matrix, provides a statistical prerequisite for the subsequent mediation analysis.

**Table 2:** Correlation analysis.

Variables	Mean (M)	Standard deviation (SD)	1	2	3	4
Physical activity	34.69	31.439	1			
Resilience	3.33	0.943	0.396^**^	1		
Cognitive flexibility	3.38	0.931	0.363^**^	0.420^**^	1	
Work engagement	3.32	0.974	0.401^**^	0.431^**^	0.435^**^	1

**p < 0.01.

### 3.3 Chain mediating effect test

Using Model 6 in the PROCESS macro developed by Hayes, a chain mediation model was constructed with physical activity as the independent variable, resilience and cognitive flexibility as the mediating variables, and work engagement as the dependent variable. The number of bootstrap resampling iterations was set to 5,000, and the confidence interval was set at 95%. Table 3 presents the regression results for this model. The path coefficients among the variables showed that when resilience was specified as the outcome variable, physical activity had a significant positive regression coefficient (B = 0.0119, *p* < 0.001, 95% CI [0.0091, 0.0147]). In the equation with cognitive flexibility as the outcome variable, the regression coefficients for both physical activity (B = 0.0069, *p* < 0.001, 95% CI [0.0040, 0.0098]) and resilience (B = 0.3243, *p* < 0.001, 95% CI [0.2280, 0.4206]) were significant. When work engagement was specified as the outcome variable, physical activity (B = 0.0066, *p* < 0.001, 95% CI [0.0037, 0.0096]), resilience (B = 0.2466, *p* < 0.001, 95% CI [0.1455, 0.3477]), and cognitive flexibility (B = 0.2691, *p* < 0.001, 95% CI [0.1682, 0.3700]) all exhibited significant positive regression coefficients. In addition, gender, age, and years of teaching were included as covariates in the model. The potential confounding effects of educational level and professional title were also examined; they were not significantly associated with work engagement in the preliminary analysis and their inclusion did not alter the significance or magnitude of the core mediation pathways. To maintain model parsimony, they were not retained in the final model presented here.

**Table 3:** Testing the chain mediation model of physical activity, resilience, cognitive flexibility, and work engagement.

Regression equation		Overall fit index			Regression coefficient				
Resulting variables	Predictor variables	*R*	*R^2^*	*F*	*β*	*SE*	*t*	LLCI	ULCI
Resilience	Physical activity	0.3956	0.1565	69.7593^***^	0.0119	0.0014	8.3522^***^	0.0091	0.0147
Cognitive flexibility	Physical activity	0.4716	0.2224	53.6235^***^	0.0069	0.0015	4.6927^***^	0.0040	0.0098
	Resilience	—	—	—	0.3243	0.0490	6.6223^***^	0.2280	0.4206
Work engagement	Physical activity	0.5481	0.3004	53.5235^***^	0.0213	0.0015	4.4030^***^	0.0124	0.0312
	Resilience	—	—	—	0.2466	0.0514	4.7952^***^	0.1455	0.3477
	Cognitive flexibility	—	—	—	0.2691	0.0513	5.2453^***^	0.1682	0.3700

**p < 0.01, ^***^p < 0.001.

**Figure 2:**
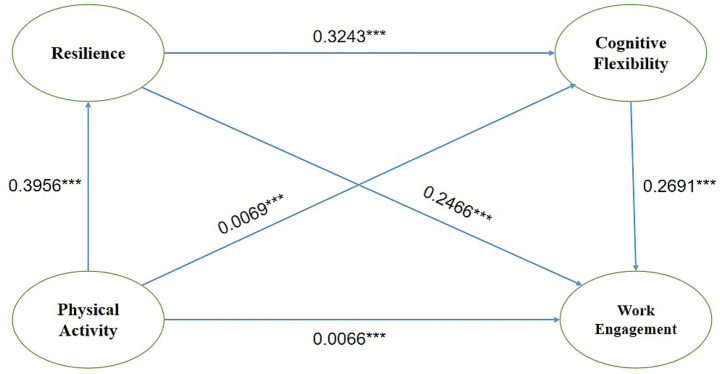
Regression analysis of the chain mediation model.

Table 4 presents the overall path coefficients from the mediation analysis. In the total effect model, the estimated total effect of physical activity on work engagement was 0.0124 (95% CI [0.0095, 0.0153]). After including resilience and cognitive flexibility as mediating variables and constructing the chain mediation model, the total indirect effect was 0.0058, accounting for 46.77% of the relative effect, with its 95% CI [0.0043, 0.0074] excluding zero, indicating that resilience and cognitive flexibility serve a chain mediating role in this model. Further decomposition revealed four pathways through which physical activity affects work engagement. Path 1 was the direct effect of physical activity on work engagement, with an effect size of 0.0066 (95% CI [0.0037, 0.0096]), accounting for 53.23% of the relative effect. Path 2 was the indirect effect of “physical activity → resilience → work engagement,” with an effect size of 0.0029 (95% CI [0.0016, 0.0075]), accounting for 23.39%. Path 3 was the indirect effect of “physical activity → cognitive flexibility → work engagement,” with an effect size of 0.0019 (95% CI [0.0009, 0.0030]), accounting for 15.32%. Path 4 was the chain indirect effect of “physical activity → resilience → cognitive flexibility → work engagement,” with an effect size of 0.0010 (95% CI [0.0006, 0.0016]), accounting for 8.06%. The 95% confidence intervals for all four pathways did not contain zero, indicating that resilience and cognitive flexibility not only independently mediate the relationship between physical activity and work engagement but also jointly function as chain mediators in this relationship (see Figure 2).

**Table 4:** Regression analysis of the chain model of physical activity, resilience, cognitive flexibility, and work engagement.

Related paths	Effect size	Boot SE	95% Confidence Interval		Proportion of relative effect
			Lower	Upper	
Total effect	0.0124	0.0015	0.0095	0.0153	100.00%
Physical activity→Work engagement	0.0066	0.0015	0.0037	0.0096	53.23%
Physical activity→ Resilience→Work engagement	0.0029	0.0007	0.0016	0.0075	23.39%
Physical activity→Cognitive flexibility→Work engagement	0.0019	0.0005	0.0009	0.0030	15.32%
Physical activity→Resilience→Cognitive flexibility→Work engagement	0.0010	0.0003	0.0006	0.0016	8.06%
Total mediating effect	0.0058	0.0008	0.0043	0.0074	46.77%

## 4 Discussion

From the cross-disciplinary perspective of positive organizational behavior and occupational health psychology, this study constructed and empirically tested a chain mediation model aimed at deeply revealing the internal psychological and cognitive mechanisms through which university teachers’ physical activity is associated with their work engagement. Overall, this study found that the association of physical activity on university teachers’ work engagement is not a single direct pathway but rather manifests as three theoretically meaningful pathways. First, physical activity has a significant positive direct predictive effect on work engagement. Second, physical activity is indirectly associated with work engagement through resilience. Third, physical activity is indirectly associated with work engagement through cognitive flexibility. More critically, this study is the first to reveal the chain mediating role of resilience and cognitive flexibility in the relationship between physical activity and work engagement. Specifically, higher levels of physical activity are associated with higher levels of resilience; resilience, in turn, is associated with greater cognitive flexibility; and these factors collectively are associated with higher work engagement. This finding not only expands research on the spillover effects of physical exercise in organizational settings but also offers a new theoretical explanation for how health behaviors outside the workplace can translate psychological resources and cognitive functions into positive outcomes in the professional sphere (Chen and Fang, 2025; Pronk, 2021).

First, the direct effect of physical activity on work engagement. This study found that university teachers members’ levels of physical activity have a significant positive predictive effect on work engagement, supporting Hypothesis 1. This result is consistent with previous findings among general employee populations and further strengthens the theoretical proposition that physical activity has cross-domain beneficial effects on positive workplace states (van Berkel et al., 2013). Using a daily experience sampling method, Li et al. (2024) found that daily physical exercise effectively predicted next-morning positive affect and work engagement, and this effect was more pronounced under high job demands. At the theoretical level, the conservation of resources (COR) theory provides a powerful explanatory framework for this positive association (Isoard-Gautheur et al., 2019). Physical exercise can be viewed as an active resource investment behavior in the non-work domain. In other words, by investing time and physical effort in exercise, individuals accumulate valuable personal resources such as physiological energy, positive affect, and self-efficacy. Because these resources are transferable across contexts, when teachers return to their academic work, the previously accumulated psychological resources can be mobilized, manifesting as higher levels of vigor, dedication, and absorption (Ünal and Ekemen, 2026). Moreover, from the perspective of triadic reciprocal determinism (Bandura, 2011), physical exercise, as a positive behavioral factor, interacts beneficially with individuals’ internal psychological states and external work environment. Regular physical activity helps break the cycle of physical rigidity and psychological burnout that results from prolonged desk-based research and sedentary teaching among university teachers (Schiavo et al., 2019). By improving physical function and emotional state, it provides a fundamental guarantee for sustained engagement at work (Abos et al., 2021). This finding suggests that for university administrators, promoting physical activity is not merely a health campaign but a strategic human resource development tool. By fostering resilience through exercise, institutions can potentially build a faculty workforce that is not only healthier but also more capable of navigating the inevitable challenges and setbacks of academic life, thereby sustaining long-term productivity and well-being.

Second, the independent mediating role of resilience. The results indicate that resilience plays an independent mediating role between university teachers members’ physical activity and their work engagement, supporting Hypothesis 2. This finding is consistent with previous research demonstrating that physical activity can effectively cultivate individual resilience traits (Melguizo-Ibanez et al., 2022). A large-sample survey found a robust positive association between physical activity participation and resilience, with resilience serving as a key mechanism through which physical activity alleviates negative emotions (Gerber et al., 2013). Physical exercise provides individuals with a “simulated training ground” for repeatedly facing and overcoming physiological and psychological challenges. During exercise, individuals must continuously cope with muscle fatigue, adjust their breathing rhythm, and engage in the willpower contest between persisting and giving up (Belcher et al., 2021). The practical implication of this finding is that when university teachers members face obstacles in their research, increased pressure from teaching evaluations, or career bottlenecks, those who have developed greater psychological resilience through long-term, regular physical exercise are more likely to view these challenges as manageable opportunities for growth rather than devastating setbacks (Brien et al., 2020), thereby maintaining or even increasing their commitment to academic work under stressful conditions (Cakir et al., 2025). In short, the psychological resilience associated with physical activity is related to a weaker erosive effect of occupational stress on work engagement.

Third, the independent mediating role of cognitive flexibility. This study also found that cognitive flexibility played an independent mediating role between physical activity and work engagement, supporting Hypothesis 3. This finding confirms that physical activity not only enhances psychological resources at the emotional and volitional levels but also positively affects individuals’ higher-order cognitive functions, which in turn translates into positive outcomes in the work context. Uhlig et al. (2023) pointed out that cognitive flexibility is an important cognitive variable linking cognitive demands in the work environment with work engagement and fatigue. The nature of university teachers’ work is characterized by high complexity and task uncertainty, requiring them to frequently and efficiently switch among multiple cognitive modes, such as lesson preparation, classroom instruction, academic writing, and administrative task handling. As a higher-order executive function, cognitive flexibility helps teachers break free from fixed thinking patterns, attempt different research perspectives or solutions when facing academic difficulties, and adjust teaching strategies promptly when encountering poor teaching outcomes (Orakci and Khalili, 2025). The facilitative effect of physical activity on cognitive flexibility may stem from multiple physiological and psychological mechanisms (Alvarez et al., 2025). Neurophysiological studies have shown that regular aerobic exercise promotes the secretion of brain-derived neurotrophic factor (BDNF) and enhances functional connectivity in brain regions such as the prefrontal cortex and hippocampus, which are important neural substrates for executive function and cognitive flexibility (Cotman et al., 2007; Erickson et al., 2011). At the psychological level, the physically and mentally relaxed state during exercise helps reduce cognitive load and anxiety levels, thereby making thinking more divergent and flexible (Byrd-Bredbenner and Eck, 2020). Therefore, university teachers with higher levels of physical activity are better able to adjust and shift their cognitive strategies with greater ease when facing complex and changing academic tasks. This sense of cognitive ease reduces the internal friction cost during work and makes it easier for individuals to experience the sense of flow and accomplishment associated with work engagement (Bakker, 2014).

Fourth, the chained mediating effect of psychological resilience and cognitive flexibility. Based on resource conservation theory and the expansion-construction theory of positive emotions, this study constructed and tested a chained mediating model of psychological resilience and cognitive flexibility, thereby supporting Hypothesis 4. Research findings indicate that physical activity is associated with work engagement not only through resilience but also through cognitive flexibility. This finding sheds light on the psychological mechanisms involved in the transition from health behaviors outside the workplace to a positive state within the workplace (Zhang et al., 2026).

Specifically, physical activity is associated with teachers’ resilience, possibly through repeated experiences of overcoming adversity. According to the broaden-and-build theory of positive emotions (Fredrickson, 2001), resilience, as a positive psychological capital reserve, effectively broadens individuals’ attention scope and cognitive horizon, providing them with more mental slack to engage in exploratory and flexible thinking under stressful conditions (Kubicek et al., 2022). Moreover, as a resource investment behavior, physical activity first accumulates the resilience resource. Higher resilience is associated with more positive emotions and lower emotional inertia, which in turn are associated with expanded attention scope and cognitive flexibility. This higher-order cognitive resource—cognitive flexibility—then ultimately translates into work engagement, a positive work-related state (Fredrickson, 2013).

In other words, when teachers possess sufficient inner strength and resilience, they experience less cognitive interference from anxiety and emotional distress when facing cognitive challenges, thereby creating a safe internal psychological environment for the deployment of cognitive flexibility (Yagan and Kaya, 2023). This enhanced cognitive flexibility, in turn, enables teachers to more effectively adapt to the high demands and fast-paced changes of academic work, maintain concentration during complex tasks, and experience greater vitality and dedication (Savas et al., 2025). Our findings regarding chain mediation are consistent with Liu’s research, which found that physical activity influences mental health through the sequence of resilience → positive coping and extended this theoretical framework to work engagement (Liu et al., 2024). This suggests a potential direction for educational administrators: physical activity programs alone may be only a starting point; if supported by future causal research, subsequent programs that target resilience and cognitive flexibility could be key strategies for strengthening the positive association between physical activity and work engagement.

## 5 Research limitations and future directions

Although this study has achieved certain results in theoretical construction and empirical testing, several limitations remain that require improvement in future research. First, this study employed a cross-sectional design. Although the chain mediation model was tested using a rigorous bootstrap method, cross-sectional data still have limitations in inferring causal relationships. Future research should adopt longitudinal designs, diary methods, or experience sampling methods to collect data at multiple time points, thereby more rigorously revealing the temporal sequence through which physical activity dynamically affects work engagement via psychological and cognitive mechanisms. Second, the measurement of physical activity primarily relied on self-report scales, which may be subject to recall bias and social desirability effects. Subsequent research could consider incorporating objective measurement tools such as accelerometers or activity trackers to obtain more accurate physical activity data. Third, the sample of this study was limited to several universities in Sichuan Province. Although the sample size met the statistical requirements, the generalizability of the findings to the national level still requires further validation through large-sample surveys across regions and university types. Fourth, unmeasured third variables (such as personality traits—e.g., conscientiousness, neuroticism—or organizational climate—e.g., perceived organizational support) may influence the observed relationship. For example, individuals with proactive personalities may be more likely to both participate in physical activities and exhibit higher levels of work engagement, which could lead to confounding in the research findings. Future research should consider including such personality and situational variables as covariates in the analysis to more rigorously test the proposed mediating model. Fifth, while the use of self-report scales for all variables is common and practical, it may lead to an overestimation of relationships due to common method variance. Future research that combines objective measures of physical activity (e.g., accelerometers) and work engagement (e.g., ratings by supervisors or colleagues) will help enhance the validity of the findings.

## 6 Conclusion

This study employed a cross-sectional design to examine the relationship between university teachers’ physical activity and work engagement, and to deeply reveal the underlying psychological and cognitive mechanisms behind this relationship. The results directly support all of the hypotheses we proposed, confirming that the positive association of physical activity on work engagement is manifested not only as a direct positive association but also indirectly through the independent mediating pathways of cognitive flexibility and resilience, as well as the chain mediating pathway of both. Crucially, this study reveals a sequential mediating pathway through which physical activity is associated with work engagement: teachers with higher levels of physical activity participation tend to report stronger cognitive flexibility, and this greater cognitive flexibility is closely associated with higher levels of resilience, with the two factors together being associated with higher work engagement.

The above conclusions offer some preliminary practical implications; however, given the cross-sectional and correlational nature of this study, these implications should be interpreted with caution. The current data do not allow for causal conclusions regarding the effectiveness of interventions. First, for university athletics departments and faculty health management agencies, organized physical activities can be viewed as a potential component of faculty occupational health promotion systems. This can serve not only as a means to improve physical health but also as a complementary strategy to enhance work performance and academic engagement, although this possibility requires validation through future intervention studies. Second, for university administrators and policymakers, creating a campus environment conducive to regular physical activity among faculty—such as providing accessible sports facilities and implementing flexible work arrangements to ensure time for exercise—could be explored as a potentially effective strategy to support faculty’s holistic professional development and academic sustainability. However, these recommendations are preliminary and should not be equated with evidence-based policy guidelines until validated by longitudinal or experimental studies.

In summary, although these practical implications are theoretically grounded and consistent with our research findings, they currently remain speculative. There is an urgent need for studies employing intervention or longitudinal research designs to test whether promoting physical activity, resilience, and cognitive flexibility is a positive predictor of teachers’ work engagement.

Future research should strive to overcome the limitations of the cross-sectional design used in this study regarding causal inference, adopting longitudinal designs or experimental intervention studies to further establish the causal relationship between physical activity and work engagement. In addition, systematically investigating the types, frequency, and intensity of physical activity that produce optimal facilitative effects on cognitive function and work engagement will provide more precise guidance for practice. Conducting in-depth studies on the mechanistic differences across teacher subgroups with different disciplinary backgrounds and career stages, as well as cross-validating the model across different cultural contexts, will also help improve the generalizability of the findings and the precision of practical applications.

## Data Availability

The datasets presented in this study can be found in online repositories. The names of the repository/repositories and accession number(s) can be found in the article/Supplementary material.
